# The feasibility of introducing rapid diagnostic tests for malaria in drug shops in Uganda

**DOI:** 10.1186/1475-2875-9-367

**Published:** 2010-12-21

**Authors:** Anthony K Mbonye, Richard Ndyomugyenyi, Asaph Turinde, Pascal Magnussen, Siân Clarke, Clare Chandler

**Affiliations:** 1 Department of Community Health, Ministry of Health, Box 7272, Kampala, Uganda; 2 Malaria Control Programme, Ministry of Health, Box 7272, Kampala, Uganda; 3 Makerere Institute of Social Research, Box 16022, Kampala, Uganda; 4 DBL-Centre for Health Research and Development, Faculty of Life Sciences, University of Copenhagen. Denmark; 5 London School of Hygiene and Tropical Medicine, London, UK

## Abstract

**Background:**

National malaria control programmes and international agencies are keen to scale-up the use of effective rapid diagnostic tests (RDTs) for malaria. The high proportion of the Ugandan population seeking care at drug shops makes these outlets attractive as providers of malaria RDTs. However, there is no precedent for blood testing at drug shops and little is known about how such tests might be perceived and used. Understanding use of drug shops by communities in Uganda is essential to inform the design of interventions to introduce RDTs.

**Methods:**

We conducted a qualitative study, with 10 community focus group discussions, and 18 in-depth interviews with drug shop attendants, health workers and district health officials. The formative study was carried out in Mukono district, central Uganda an area of high malaria endemicity from May-July 2009.

**Results:**

Drug shops were perceived by the community as important in treating malaria and there was awareness among most drug sellers and the community that not all febrile illnesses were malaria. The idea of introducing RDTs for malaria diagnosis in drug shops was attractive to most respondents. It was anticipated that RDTs would improve access to effective treatment of malaria, offset high costs associated with poor treatment, and avoid irrational drug use. However, communities did express fear that drug shops would overprice RDTs, raising the overall treatment cost for malaria. Other fears included poor adherence to the RDT result, reuse of RDTs leading to infections and fear that RDTs would be used to test for human immune deficiency virus (HIV). All drug shops visited had no record on patient data and referral of cases to health units was noted to be poor.

**Conclusion:**

These results not only provide useful lessons for implementing the intervention study but have wide implications for scaling up malaria treatment in drug shops.

## Background

In Uganda, malaria is highly endemic and the leading cause of morbidity and mortality [1]. Effective case management of malaria is recommended as one of the interventions to control malaria. However, this is limited by inadequate diagnostic facilities. Thus the syndromic approach is the commonest form for diagnosing malaria and other infectious diseases in Uganda [2-4].

The dilemma of malaria diagnosis and treatment has been well described in Uganda and Tanzania [4]. It has been shown that more than 90% of malaria patients who are negative for malaria were prescribed anti-malarial drugs. Based on this and other findings, it has been concluded that there is over diagnosis of malaria among patients presenting with febrile illnesses [5,6].

Self-treatment is the commonest form of treatment in rural areas of Africa [7]. A recent study in Uganda found out that 67% of children aged less than 5 years suffered from malaria and that 38.3% of households sought care from drug shops for sick children [8]. It was further shown that people valued laboratory investigations commonly referred to as *okukebela omusaayi*. Health facilities with laboratory facilities were visited when self-treatment had failed and people were in search for the cause of fever and effective treatment [8].

There are various reasons why people choose to use pharmacies and drug shops. First, is the ease of purchasing drugs and obtaining *"immediate" *treatment. In a study in Togo, it was found that immediate attention for both consultation and treatment was the single most important reason for patronizing retail pharmacies rather than health clinics [9]. Another important factor was *"availability of drugs at all times," *since the pharmacy was open during hours when the health clinics were closed.

Nevertheless, self-treatment of malaria is associated with many potential dangers. First, self-treatment may result in delays in taking patients with severe malaria to health centers [10]; secondly, there is the risk of overtreatment and overdosing which may lead death while under treatment may lead to drug resistance [11-14].

The current practice of presumptive treatment of any febrile illness as malaria in Uganda results in significant over-use of anti-malarial drugs. However, with ACTs being a more costly regimen, it is important to be more restrictive whilst ensuring access to those who need anti-malarial drugs. RDTs provide a simple means of confirming malaria diagnosis in remote locations lacking electricity and qualified health staff. However, there is no precedent for blood testing at drug shops and little is known about how such tests might be perceived and used. An understanding of the current situation of drug shop attendants and the use of shops by communities in Uganda is essential to inform the design of interventions to introduce RDTs.

We present data from a formative study that assessed the feasibility of introducing RDTs in drug shops in Mukono district, central Uganda. This formative research is nested within a wider intervention study that will introduce RDTs into drug shops in a randomized controlled trial that will assess the impact of RDTs in drug shops on effective malaria treatment and rational anti-malarial drug use as well as cost-effectiveness of this intervention.

## Methods

### Study area and population

The study was conducted in Mukono district, central Uganda. The total population of the district is 850,900 and the majority, 88% live in the rural areas. The district has an annual population growth rate of 2.3% per annum and is inhabited by the Baganda an indigenous ethnic group whose main occupation is subsistence agriculture [15]. The district was selected to implement the study because it is hyper-endemic for malaria [16].

### Study design

The study design was qualitative and collected data through focus group discussions (FGDs) and in-depth interviews (IDIs). Ten FGDs were conducted as follows: four female groups, three male groups and three community leader groups. Each FGD comprised of 6-12 participants. A total of 18 in-depth interviews were conducted with nine drug shop attendants, five health workers and four district health officials (Table 1). The FGDs and IDIs explored the following areas: experiences with diagnosis and treatment of malaria, affordability of malaria treatment and RDTs, quality of care at drug shops and payments for services at drug shops.

**Table 1:** Sample sizes for the study and background characteristics of respondents

Type of Sample	Sampling technique	Sample size
Focus Group Discussions (FGDs)	Purposively selected; males and females with children aged< 5 years.	10 FGDs
		
In-depth Interviews (IDIs)	Purposively selected; targeting civic leaders, local council officials, drug shops sellers and health workers	18 KIs
		
**Socio-demographic characteristics**		
Age range (FGDs-females)	(18-52 years)	
Age range (FGDs-males)	(24-64 years)	
		
Average number of children < 5 years (FGD participants)	2 (minimum = 1, maximum = 4)	

Participants for the FGDs and IDIs were selected with the help of local council officials who were instrumental in introducing interviewers to the communities. The inclusion criteria were respondents in the reproductive age group with children aged < 5years and consent to participate in the study. The exclusion criterion was refusal to participate in the study.

FGDs and IDIs were conducted by two female and two male research assistants who are social scientists with experience in conducting qualitative research. All participants spoke *Luganda*, the local dialect, and underwent training specifically in data collection methods and tools to be used. The study tools were piloted and revised. FGDs and IDIs were tape-recorded with permission from the participants. The study was conducted between May-July 2009.

### Data analyses

Data were transcribed and transferred to NVivo version 8 (QSR International). Coding of the transcripts took place through an iterative process to generate a 'node tree' of ideas represented in the data that were grouped into themes. The themes were initially drawn from the idea codes.

### Ethical issues

The study was approved by the Uganda National Council of Science and Technology Reference: HS 546. Verbal consent was obtained from all participants who participated in the study.

## Results

### Role of drug shops

Community members, health workers and the district health officials acknowledged the importance of drug shops play in providing health care. Our study focused on registered drug shops and respondents did distinguish these from non-registered shops, which were perceived to be numerous and to provide a poorer quality of care. The typical interaction described at registered drug shops was as follows:

*'Before you get the drug the one working there first asks you what the problem is before getting the drugs and then gives you the drugs basing on the money you have' *(Participant 3, FGD with community leaders at community 9).

### Current diagnosis and treatment practices

The most common methods of malaria diagnosis to occur at registered drug shops were syndromic and trial-and-error approaches. Occasionally, community members and drug sellers reported that drug shops in the study area used thermometers.

*'For me when I reached there at the drug shop, my temperature was measured using a thermometer. The drug seller told me that I had fever though she didn't tell me the type of fever I had, so she told me that I was going to take tablets and injections and see whether it would help me; and if it doesn't, I then go elsewhere*' (Participant 1, FGD with fathers at community 3).

In addition, there were rare reports of drug shops requesting patients to go for laboratory investigations before giving treatment, mostly after a patient had earlier received treatment but not improved. In one drug shop, it was reported that a "strip" was used to diagnose malaria.

From discussions with district health officials and in-charges of health units, it was acknowledged that diagnoses of malaria at health facilities were based on the syndromic approach. At public health facilities with laboratory services, diagnosis of malaria was constrained by inadequate laboratory equipment especially microscopes, reagents and other laboratory supplies. The other major constraint was heavy patient load.

*'The main method used to diagnose malaria is by clinical impression, because health workers have a heavy client load and can't send every febrile patient to the laboratory' *(District Health Official 3).

The syndromic approach to diagnose febrile illness was promoted by senior health workers. In fact one of the district health officials noted that this approach is most appropriate in areas where malaria is endemic.

'*Malaria is the commonest cause of fever within our setting because even under the home based management of fever, which is recognized by the Ministry of Health, it is clearly indicated that any fever is malaria unless proved otherwise*' (District Health Official 3).

### Perception of laboratory diagnoses

Although laboratory based microscopic diagnoses was rarely sought, it was commonly reported that patients resorted to it after failed presumptive treatment. The concept of testing was highly appreciated by patients. This was reflected by high awareness amongst all patient groups that fevers are not always malaria.

*"In fact most of the time I go to a health unit, I like to be tested because I get to know what the problem is so that they treat the real cause. Because you may think that it is malaria, when it is actually another type of fever such as typhoid. So I like to test blood most of the time" *(participant 2, FGD with community leaders at community 6).

*'Malaria is known to share symptoms with other illness, say cough or a wound. Such illness you may think its malaria and because there are IMCI meant to treat children, there they tell us that any person with a high temperature should be administered with a malaria treatment. But it would be proper to test blood and be sure that it's real malaria*' (Health worker 5, enrolled midwife at health centre B).

In general, laboratory results were perceived to be accurate, although trust in results was highly dependent on the outcome of treatment.

*'When they test you and get the treatment and you become fine, there you become fine, there you believe the tests. But if you do not become fine, you may doubt the tests carried out' *(Participant 4, FGD with community leaders at community 6).

### Perceived benefits of RDTs in drug shops

Most community members and drug sellers did not know about RDTs for malaria diagnosis; but after they received an explanation, they felt it would be a useful intervention. Health officials also saw the advantage in convenience and more accurate treatment,

*'If they are trained I think it is ok it will make it like in the health units and it will make it convenient. They will not treat blindly. I think the idea is ok' *(District Health Official 2).

### RDTs may increase confidence in treatment

Building on perceptions that laboratory investigations for malaria facilitate access to effective treatment, respondents anticipated that RDTs would reduce what was referred to by both recipients and providers as *"blind treatment" *or *"guess work"*. It was perceived that the use of RDTs would help to establish the cause of illness, which would enable providers to give effective treatment.

*'I welcome RDTs with much interest... Because a patient can come with fever but claiming to be malaria but when actually it is flu which has caused that, do you see? But when you test and the result is negative, you cannot go ahead to spoil the drug which you could have given another person with malaria. Do you see? Now you give another drug which treats the expected illness as you explain to the patient that 'you don't have malaria but the fever is due to the flu you have this'... and the person gets well, becomes happy and will have used little money. Because spending 6,000/= on Coartem when not treating malaria, this is a great loss to the patient!' *(Drug Shop Seller 7).

*'Now these people and also we [ourselves] have just been guessing that it might be malaria but with this test, it will confirm that it's either malaria or not' *(Participant 4, FGD with fathers at community 3).

### RDTs may boost confidence and business for drug shops

The presence of RDTs was seen as beneficial to all drug shop attendants interviewed, who saw that the tests would improve their reputation and attract patients.

*'My business will be moving much better than before because people will be saying that "Musawo [doctor] tests your blood first and knows what exactly the problem is and then prescribes the treatment accordingly"' *(Drug Shop Seller 8).

A direct impact on business was anticipated, through increased numbers of patients and the probability that patients testing positive with RDTs being unable to refuse to purchase anti-malarials. One drug seller described how he might manipulate the price of RDTs as he has done for drugs, in response to prices of other shops as well as once his own reputation was good,

*'Now if you treat people well, and talk to people well, [they will] always say that the health worker treats people well, even if she gives you only two tablets, you become fine. So when I hear such statements, I may increase the price for the dosage' *(Drug Shop Seller 4).

Another drug seller explained that, rather than charging for the test and drugs separately, he would combine the two costs into a 'service' charge,

*'It will be possible for them to pay for both the RDT and the drugs and very easy because when they come I will be telling them only the amount of the services but not telling them to pay for the RDT separately from the drugs' *(Drug Shop Seller 5)

### RDTs may save money and time for clients

Besides improving effective treatment for malaria, drug sellers also thought that RDTs would reduce unnecessary use of drugs and this would save money for clients. For example, diseases such as cough, flu and pneumonia present with high fever like malaria but with no diagnostics such diseases are treated as malaria, leading to drug wastage and inappropriate treatment.

Drug sellers perceived RDTs as time saving and leading to a quick service to clients. When drug sellers do refer patients for microscopy this only delays treatment but is also costly as patients have to incur transport and other costs.

*'RDTs will make work easier in that time wasted in sending a patient for blood testing... all this time will now be put to serving patients and in one place thus time saving' *(Drug Shop Seller 5).

### RDTs may promote referral

There is no formal system for referral from drug shops to health facilities. However, respondents in our study suggested that the use of RDTs might encourage referral by drug sellers for patients with negative test results,

*"RDTs will be very useful because they help to know what the patient is suffering from, instead of giving drugs without knowing whether it's malaria or not. You also get to know whether you can handle the case or should refer the patient" *(Drug Shop Seller 4).

However, many drug shop sellers interviewed reported unease about referring, citing experience that patients were reluctant to be referred due to transport costs, long waiting times and poor treatment at health centres.

### Potential challenges of introducing RDTs in drug shops

#### RDTs may increase cost of visit

A few community members feared that drug shops would hike the charges for RDTs because they want to reap as much profit as they can. Respondents observed that already many of the patients could not afford to pay for drugs. There were fears that some patients may not seek care at drug shops since the cost of RDTs would increase the overall treatment costs.

*'Now, as you know, these health workers who put up drug shops want money, so when you go there to test your blood, he will say this, "eh, today the test that has been at 2,000/= has increased to 5,000/= because this one is too fast and quick, and I don't want you to say that 'Musawo [doctor] has increased the price' but because am testing you"' *(Participant 4, FGD with mothers at community 1)

*'They will also tell us that eh this thing is expensive but it detects malaria so fast, so if you want to go quickly, bring more money and we test you and go now!' *(Participant 5, FGD with mothers at community 1)

District officials were concerned that the additional costs of RDTs might in fact decrease access to treatment if patients were deterred by the costs at drug shops,

*'You see drug sellers on top of providing a service, they are also interested in making profits so any medical intervention we must weigh how will it affect the flow of the patients to the drug sellers. Most of our people are poor... so if it is going to add on the expense the consumption level may be low. So we need to weigh, is it going to reduce the profits or is it going to deter clients?' *(District Health Official 3)

#### RDTs may not lead to correct treatment

Another fear expressed was that laboratory tests carried out in private clinics are not always accurate partly because private providers are perceived to be money-minded and by extension may treat patients with RDT negative results with anti-malarial drugs. In addition, respondents noted that RDTs only test for malaria whilst many types of fever were known to exist, with frequent references to typhoid and yellow fever.

*"The patient can present with a fever which may not be malaria yet the RDT only detects malaria" *(a health worker at Kasawo health centre).

*"For me, my only problem with that thing [RDT] is that, it shows only type of fever. It does not differentiate between the type of fevers" *(Participant 3, FGD with adult men at community 4).

#### RDTs may lead to increased client risks

There was fear that RDTs used at drug shops could be used to test for HIV when people have not consented to know their status. This may lead some clients to refuse to be tested, as one drug shop seller described in the case of sending for microscopy (blood slide) testing,

*'Uhm but some do refuse because there are those who do not want to test for HIV/AIDS, so when you advise them to go for a blood slide they mistake this for HIV testing and such people will never accept to be tested however much you explain' *(Drug Shop Seller 5).

*"There is need for sensitization because in most communities people think that taking of blood means testing for HIV and some don't want their HIV status disclosed. Because of this, some people may shy away from the drug shops. The truth is that people will ask many questions about testing with RDTs, but with time, through sensitization, they will get to understand" *(Health Worker 4, in-charge at Health Centre C).

There was also fear that some drug shops may not observe good hygiene practices while performing tests and, therefore, could transmit infections. Some participants reported that some drug sellers at times give treatment to clients with dirty hands.

#### RDTs may not lead to improved referral

There was a concern among community members and health workers that in spite of the use of RDTs at drug shops, referral may still not occur, with frequent reports that drug shop sellers prefer to continue to treat patients than refer, even when seriously ill.

*'Once you enter into a drug shop even when the illness is beyond them, they can't leave you to go, they make sure that they get some money out of you' *(Participant 3, FGD with community leaders at community 9).

*'I think it's because they don't want to miss money. So they try their best to treat the patient, till the patient sees no sign of improvement and decides to go to a government hospital on his/her own' *(Health Worker 2, nursing assistant at Health Centre B).

#### RDTs may be carried out by unqualified staff

This study sought to find out the extent to which different actors trusted drug shops. A problem cited by many health workers and officials as well as community members was the lack of trained staff attending at drug shops.

*"The problem we have with drug shops is that some of the people who run them are not fully qualified. Drug shops are registered by qualified personnel who in turn recruit less qualified people to run them on their behalf." *(District Health Official 1).

*"Most of the drug shops here are operated by unqualified people. I have a live example, I went to a drug shop I had taken my grandchild for treatment they gave us some tablets which were meant for adults. After giving the tablets to the child he started vomiting blood" *(Participant 6, FGD with community leaders at community 6).

None of the drug shops visited had treatment guidelines or charts visible as reference materials. Drug sellers reported that in case they wanted to consult, they rely on text books, other drug sellers or public health workers.

Drug sellers were asked how they get information about new treatment regimes. They said they get information either through the radio, when they have visited public health facilities and from the information sheets inside drug packets. Through such sources, all drug sellers interacted with during the study knew that Coartem was the first-line treatment for malaria.

#### RDTs may suffer from lack of supervision and support from the district

This study noted inadequate supervision of drug shops because there was only one officer in the district who supervises drug shops.

*"One of the biggest challenges we have in the district is the supervision of *drug shops*. We need more personnel to assist with supervision when RDTs are introduced. For instance, in the district, there are over 300 drug shops and you have one person to supervise all of them, it is a very big task" *(District Health Official 3).

Although drug shops were perceived to play an important role in treating malaria; this study found that there was little support from government. For example numerous workshops were held in the district to refresh public health workers, excluding drug sellers.

*"Normally, when workshops are organized the drug shop attendants are not invited. Yet most people running these shops are not trained and there is need to train them. Another problem is that even when the district distributes new materials, drug shops are not considered, like the recent distribution of Uganda Clinical Guidelines" *(Health Worker 4, in-charge at Health Centre C).

In addition, the study found out that there was poor record keeping at drug shops, likely a reflection of their lack of inclusion in the public health system, and with the result of making it difficult to evaluate practices.

## Discussion

The present study shows that introducing RDTs for malaria diagnosis in drug shops was highly accepted; although people in this community did not have prior experience with RDTs. RDTs were perceived to improve access to effective treatment of malaria, reduce costs associated with poor treatment, and irrational drug use. However, it perceived a risk of introducing RDTs due to increased cost of the test, potentially forcing clients to seek care elsewhere, a potential delay to referral and a lack of integration with the public health system which would not support supervision and evaluation of RDT use.

Scaling up RDTs for effective treatment of malaria is now a priory intervention in Uganda. This is initiative is supported by recent findings indicating that the current clinical practice in Uganda results in massive over-diagnosis across all age groups with over treatment of malaria [17]. Introducing RDTs is also based on studies elsewhere that have shown that RDTs are highly sensitive in diagnosing malaria [18-20]. In addition, RDTs have been found to be cost-effective in diagnosing malaria in out patients in Zambia [21].

The pricing of RDTs in drug shops remains a major challenge and our results suggest that clients are already suspicious of profit-centred motives of drug shop attendants which may be increased by introduction of RDTs. Suggestions of fixed prices, subsidized prices and free-marked prices were made and each of these needs to be explored carefully through trials.

Treatment-seeking behaviour and perceptions on laboratory investigations related to malaria in pregnancy has previously been documented in the study area and the stigma related to HIV infection among pregnant adolescents was found to be a barrier to seeking investigations for malaria [8]. These findings highlight the need to train drug sellers to explain how the RDTs work and that they specifically test malaria not HIV. It is important that whenever clients get in touch with service providers, counseling on common illnesses like HIV infection is done, stigma dispelled and referrals made if possible. We recommend further research to identify modalities for integrated counseling on common illnesses.

In a study of home treatment of febrile children in Togo, it was found that 83% of children were treated with an anti-malarial drug at home [9]. In a review of the treatment-seeking for malaria, [11,12] it was found that care for most malaria episodes begins with self-treatment, and that nearly half of all cases were self-treatment. It has been argued that were people in many developing countries to cease self-treatment and seek care at health facilities, these institutions would be unable to cope with the large influx of patients [10].

This study underscored that one of the apparent fears to introduce RDTs in drug shops was non-adherence to the test result and fear that RDTs may test for human immunodeficiency virus (HIV). Similar findings on poor diagnosis of malaria have been documented in Tanzania, where malaria diagnosis did not follow set guidelines but was influenced by the mind-set of clinicians [22]. Similar fears for HIV testing and poor quality control of RDTs have also been documented in Tanzania [23,24]. It will be important to train drug shop attendants to adhere to the RDT result and the importance of this for effective fever treatment.

Our results show that drug shop attendants have an uneasy relationship with the public health system as well as with regulatory authorities. A system for supervision of these workers is urgently needed if they are to be included in the fight for improve fever case management. Supportive supervision will be important in ensuring tests and results are used correctly in practice. In the main intervention, the sub-county trainers and the district assistant drug inspector (DADI) will provide supervision to drug shops. A social scientist will conduct a continuous evaluation of this aspect to provide useful lessons for sustainability of this intervention.

Referral is an important part of this study. By law, registered drug shops are not permitted to stock antibiotics or other "prescription only" medicines. Commercial drug sellers will therefore willbe trained to examine and refer febrile patients with a negative RDT (i.e. non-malaria) to the nearest health facility for examination and treatment by a fully qualified health worker, where appropriate. Any patient with danger signs and/or a temperature > 38.5 will be referred to the nearest health unit as an urgent case. This will be evaluated and lessons obtained for future strengthening of referral of patients.

We found that many drug shop attendants were not qualified health workers. In order to introduce RDTs into drug shops, it will be necessary to train the drug sellers to equip them with skills and knowledge on how the RDTs work so that they give appropriate explanations to clients. The research team together with the DADI will provide close supervision so at to provide useful lessons for improving treatment of malaria in drug shops.

It will also be necessary for communities to be sensitized about the test, including addressing fears over hygiene, HIV and costs. The sensitization could utilize local channels such as social gatherings, churches, political meetings and local radio talk shows.

The perceptions and acceptability of RDTs in this present study are based on populations' experience of treatment seeking, including the use of laboratory investigations of malaria and other illnesses. Although this is useful in anticipating potential benefits and risks of the approach, it will also be important to document how the RDTs are used in practice. Intensive evaluations are required to document the population's experiences with RDTs over time, including how community and provider perceptions of RDTs interact with the use of the tests and of anti-malarial drugs as well as wider potential impacts of RDT implementation in shops. These should include analysis of impact on individual and household behaviour, provider behaviour and community-provider relationships. Furthermore, impacts on the public health system and on the private sector should be measured. A detailed framework for this analysis has been developed by the ACT Consortium [25].

## Conclusions

Our study suggests that introducing rapid diagnostic tests for malaria into drug shops is feasible. Community members and drug sellers perceived RDTs as a useful strategy for effective treatment of malaria. However, we identify a number of challenges that will need to be addressed if RDTs are to be used effectively, including guidelines for treatment or referral of RDT negative patients, regulation or subsidization of the price of RDTs and a system for training and supervising drug sellers to use RDTs and adhere to results. These challenges cannot be underestimated given the important but neglected position of drug shops in the public health system in countries such as Uganda.

## Competing interests

The authors declare that they have no competing interests.'

## Authors' contributions

AKM was the principal investigator, conceptualized this sub-study and drafted the manuscript with CC while RN, PM and SC are co-investigators and contributed to the study design, and reviewing the manuscript. CC and AT participated in design of study tools, data collection analyses, drafting and reviewing the manuscript. All authors read and approved the final manuscript
